# Neuroprotection by IFN-γ *via* astrocyte-secreted IL-6 in acute neuroinflammation

**DOI:** 10.18632/oncotarget.16990

**Published:** 2017-04-09

**Authors:** Lijie Sun, Yan Li, Xiuzhi Jia, Qi Wang, Yue Li, Minghui Hu, Linlu Tian, Jinfeng Yang, Wenjing Xing, Weihua Zhang, Jingtao Wang, Hongwei Xu, Lihua Wang, Dekai Zhang, Huan Ren

**Affiliations:** ^1^ Department of Immunology, Harbin Medical University, Harbin, China; ^2^ Key Laboratory of Infection & Immunity, Heilongjiang Province, Harbin, China; ^3^ Center for Infectious and Inflammatory Disease, Institute of Biosciences and Technology, Texas A&M University System Health Science Center, Houston, TX, USA; ^4^ Department of Clinical Laboratory, The Affiliated Hospital to Qingdao University, Qingdao, China; ^5^ Department of Pathophysiology, Harbin Medical University, Harbin, China; ^6^ Department of Epidemiology and Biostatistics, The Public Health Institute, Harbin Medical University, Harbin, China; ^7^ Department of Neuroscience, The Second Hospital Affiliated to Harbin Medical University, Harbin, China

**Keywords:** interferon-gamma, astrocyte, interleukin-6, apoptosis, neuroinflammation, Immunology and Microbiology Section, Immune response, Immunity

## Abstract

Inflammation eliminates pathogenic infections while also threatening the integrity of the central nervous system. In this study, using *in vivo* and *in vitro* models of acute neuroinflammation, we investigated the mechanisms by which inflammation and astrocytes affect neuronal apoptosis. The *in vitro* model mimicked acute neuroinflammation by incubation in IFN-γ-containing media with primary cultured cerebellar granule neurons, with or without cultured astrocytes. This quickly induced neuronal apoptosis characterized by cleaved caspase-3 expression, Hoechst 33342 staining, and intercellular Ca^2+^ influx, whereas the presence of astrocytes significantly protected neurons from these effects. IFN-γ in the inflammation media also promoted astrocyte secretion of IL-6, essential for protection. The supernatants of rat peripheral blood mononuclear cells stimulated by lymphocyte mitogen lipopolysaccharide or concanavalin A were used as inflammation media to verify the results. The *in vivo* model involved a peripheral challenge with lipopolysaccharide, with or without recombinant IFN-γ, in C57BL/6 mice. This confirmed the *in vitro* results: anti-IFN-γ antibodies exacerbated the acute course of neuroinflammation and led to neurocyte apoptosis *in vivo*. The pro-inflammatory cytokine IFN-γ provided neuroprotection during acute neuroinflammation via induction of astrocyte-secreted IL-6. The findings provide novel insights into the mechanisms of neuroprotection by IFN-γ during acute neuroinflammation, and may impact therapies for inflammation-related central nervous system injury and disease.

## INTRODUCTION

Immune privilege is an intrinsic property of the central nervous system (CNS) [1, 2]. Although inflammation is required for eliminating pathogenic infections, extremely intense or chronic inflammation can threaten the integrity and function of the CNS. Increasing evidence shows that immune responses and inflammation are important in CNS injury and disease [1, 2]. However, the mechanisms by which neuronal and supporting tissues regulate inflammation to maintain homeostasis and immune privilege are not fully understood. During inflammation, the CNS can release large numbers of inflammatory mediators, including neurotransmitters, cytokines, chemokines, and active oxygen free radicals. An uncontrolled inflammatory response is toxic to neurons and causes diseases such as Alzheimer's [3, 4], Parkinson’s, and multiple sclerosis, as well as trauma and infection, via the local activation of different types of resident cells [5, 6].

Pro-inflammatory cytokines, such as IL-1β, TNF-α, and IL-6, have multiple roles in both neurodegeneration and protection. These acute-phase response proteins contribute to the development and resolution of acute and chronic inflammation [7]. IL-6 is a pleiotropic cytokine produced mainly by activated microglia and astrocytes in different brain regions; it is associated with protective CNS functions via promotion of neuronal survival and regeneration [8, 9]. Astrocytes, in addition to secreting IL-6, also actively regulate the microglia at multiple levels during pro-inflammatory injury and repair. Inhibited IFN-γ expression in astrocytes causes sustained neurological disability, which correlates with increased levels of TNF-α and IL-6 [10]. Research has shown that IFN-γ induces neuronal damage during chronic inflammation [11]; however, other reports have indicated a neuroprotective role for IFN-γ, associated with immune-mediated mechanisms [12–14]. Collectively, these reports imply that inflammatory cytokines may mediate opposite effects based on the specific situation. Currently, the immunological mechanisms involved in protecting neurons from biological challenges remains to be fully elucidated.

CNS inflammation is often initiated peripherally, i.e. via severe bacterial or viral infections. Such acute inflammation can produce a large number of cytokines and chemokines toxic to neurons, while also destroying invading pathogens [1, 2]. In the present study, to mimic certain situations and elucidate the relevant mechanisms, we used inflammatory-condition media containing the supernatants of lipopolysaccharide (LPS) or concanavalin A (ConA)-stimulated peripheral blood mononuclear cells (PBMCs) in primary cultured rat neurons in the presence or absence of astrocytes. LPS and ConA are mitogens that non-specifically stimulate T and B cells, activation of which can quickly produce abundant pro-inflammatory cytokines, including IFN-γ and IL-6, as in the active immune response [11]. In such an inflammatory environment, the activation of astrocytes via the release of additional cytokines into the buffer protects neurons while also sustaining the local inflammatory environment for a certain period of time. In addition, we modeled acute CNS inflammation with C57BL/6 mice *in vivo* via peripheral injection of LPS with recombinant IFN-γ to confirm the *in vitro* data. The results showed that anti-IFN-γ antibody treatment delayed resolution of acute inflammation, and IFN-γ was responsible for neuroprotective IL-6 secretion by activated astrocytes. Our data elucidated some of the mechanisms of the inflammatory environment and astrocytes with regard to neuronal apoptosis, which may provide a novel strategy to prevent neuronal damage during inflammatory CNS injury and disease.

## RESULTS

### Astrocytes protect neurons from acute inflammation-induced apoptosis

We recently developed and optimized conditioned media for feeding neurons, to establish an experimental neuroinflammatory environment [11]. To determine how this acute inflammatory environment affects neurons and to investigate the role of the pro-inflammatory cytokine IFN-γ, we incubated primary cultured cerebella granule neurons (CGNs) in inflammation media derived from supernatants of PBMCs of Wistar rats under either LPS or ConA stimulation [11]. The conditioned media were taken from 24 h stimulation by LPS or ConA, as the levels of IFN-γ in the inflammatory milieu were relatively high (Figure S1A and S1B; Table S1 and S2). We named the respective media LPS-CM and ConA-CM. Of note, the level of IFN-γ in ConA-CM was approximately half of that in LPS-CM (Figure S1A and S1B).

**Figure 1:**
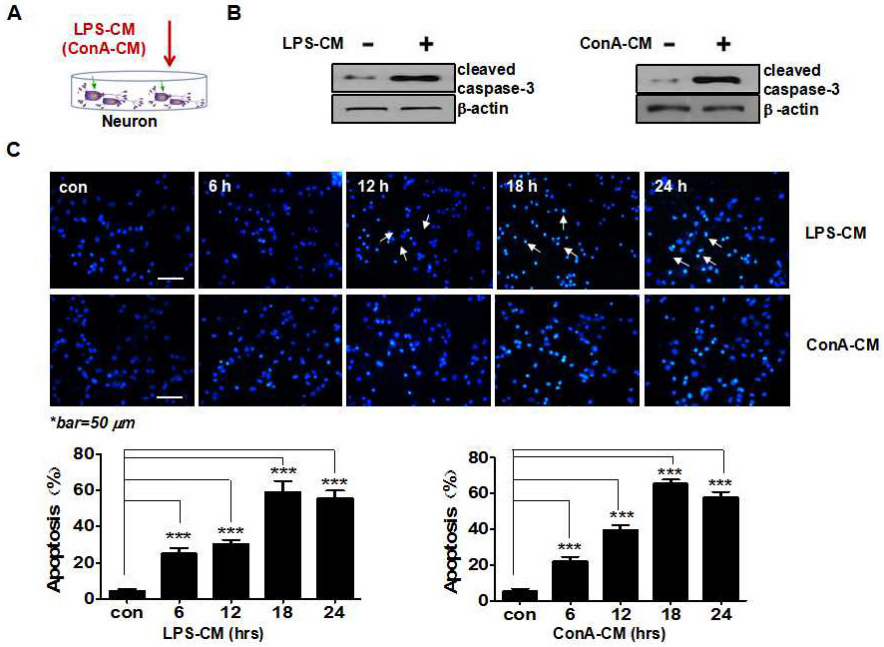
Acute inflammatory environment induces neuronal apoptosis **A**. Schematic representation of the experimental model with inflammation medium to challenge neurons. **B**. LPS-CM or ConA-CM stimulation induces the expression of cleaved caspase-3. **C**. The primary cultured neurons (CGNs) were incubated with LPS-CM or ConA-CM for 6, 12, 18, or 24 h. Neuronal apoptosis was determined with Hoechst staining and quantified with NIH Image J. Values represent the group means ± SEM for four individual experiments. *Typical apoptotic nuclei (condensed, fragmented, bright white) in contrast to non-apoptotic nuclei (non-condensed, blueish) were only partially sampled with arrows starting from 12 h with LPS-CM stimulation. ***P < 0.001 vs. control group.

We first investigated how LPS-CM and ConA-CM affect neuronal apoptosis by checking the caspase-dependent apoptotic pathway (Figure 1A and 1B). At 24 h post-LPS-CM or ConA-CM stimulation, the expression of cleaved caspase-3 in neurons was significantly increased (Figure 1B). Both inflammatory media rapidly induced neuronal apoptosis in the primary cultured CGNs in similar patterns during incubation. Hoechst staining showed that the level of neuronal apoptosis peaked 18 h post-incubation, when apoptosis had been induced in approximately 58.13% and 63.29% of the neurons exposed to LPS-CM and ConA-CM, respectively (Figure 1C). In contrast, neither LPS-CM nor ConA-CM stimulation induced cell death in the primary cultured astrocytes (data not shown).

**Figure 2:**
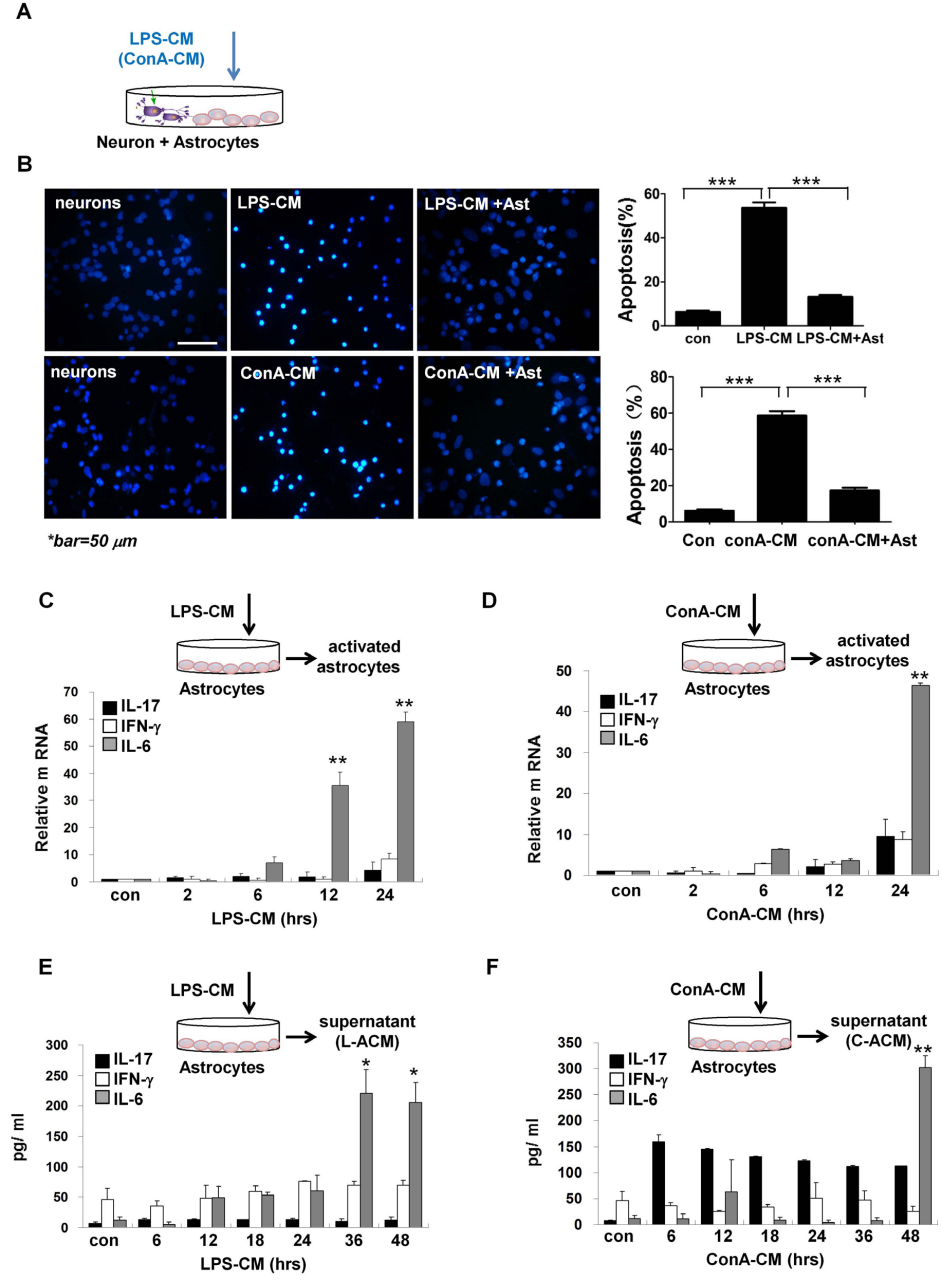
Astrocytes secret IL-6 and protect neurons under inflammatory stimulation **A**. Schematic representation of the experimental model with inflammation medium to challenge the co-cultures of primary culture CGNs with astrocytes. **B**. Astrocytes protect neurons from apoptosis against LPS-CM or ConA-CM stimulation. ***P < 0.001 vs. LPS-CM or ConA-CM group. The primary culture astrocytes were incubated with LPS-CM or ConA-CM for different times. The mRNA of IL-17, IFN-γ, and IL-6 was determined with qRT-PCR **C**., **D**. and their protein levels in the supernatant were measured with ELISA (**E**., **F**). **P < 0.01 vs. control group. Value represent group means ± SEM for four individual experiments.

We next applied LPS-CM or ConA-CM to co-cultures of CGNs and primary culture astrocytes from Wistar rats, and stored them for up to 48 h (Figure 2A). We found that the presence of astrocytes led to greatly reduced rates of neuronal apoptosis under the inflammation stimuli (Figure 2B), indicating that astrocytes can protect neurons from apoptosis under acute inflammatory conditions.

### IL-6 produced by activated astrocytes is neuroprotective

We assumed that the astrocytes protected neurons via the secretion of neuroprotective factors. To test this hypothesis, we incubated the primary cultured astrocytes with LPS-CM or ConA-CM and analyzed the activated genes in the astrocytes and secreted factors in the supernatants (Figure 2C-2F). Notably, IL-6 was readily induced during the incubation at both the mRNA level (Figure 2C and 2D) and the protein level (Figure 2E and 2F). ConA-CM also induced a considerable amount of IL-17 in the astrocytes during incubation based on ELISA assays (Figure 2F), consistent with our previous finding that IL-17 was a neuroprotective factor during acute neuroinflammation [11].

**Figure 3:**
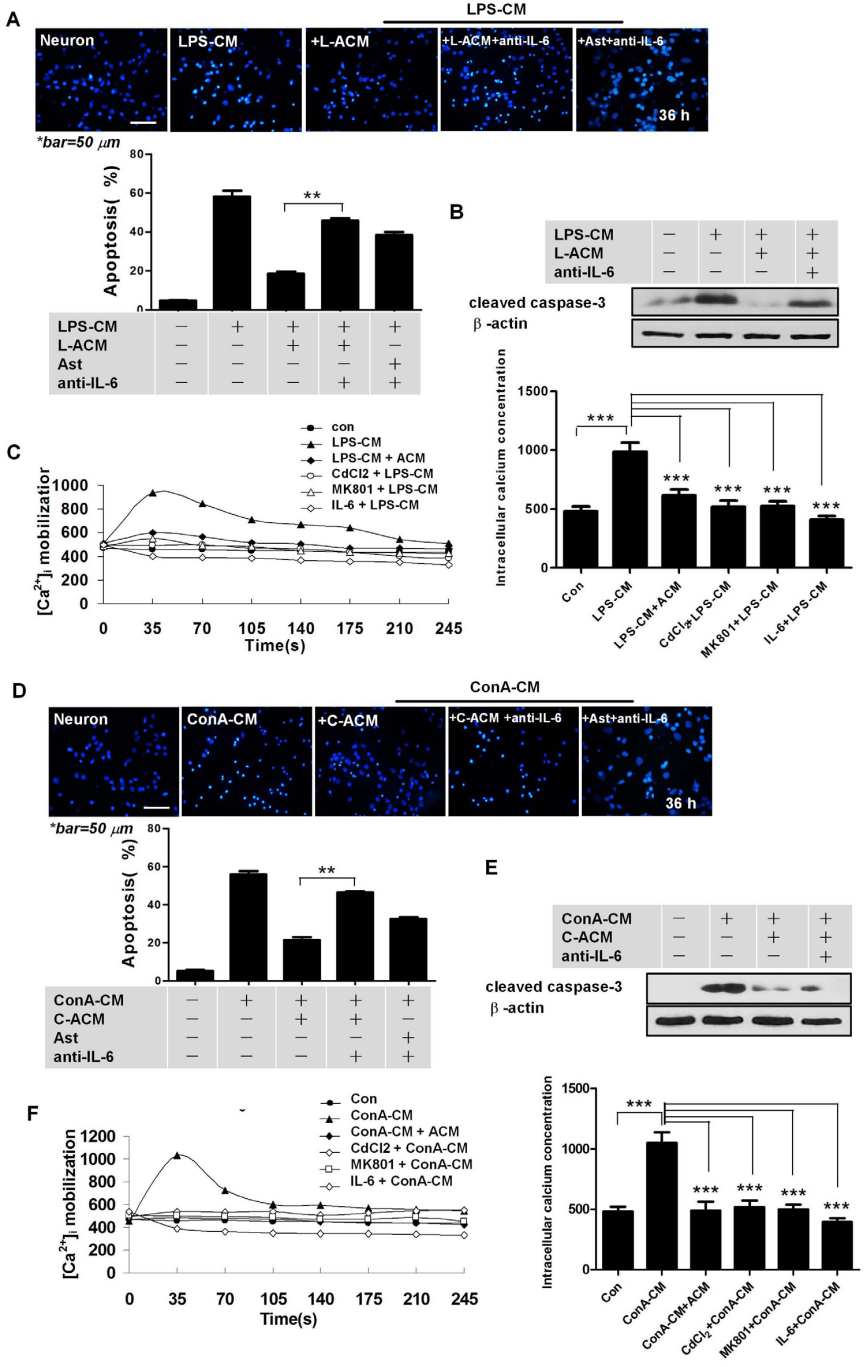
IL-6 is important for astrocyte-mediated neuroprotection **A**., **D**. Anti-IL-6 antibodies abolished the neuroprotective effects of astrocytes, or of L-ACM (or C-ACM) on neurons under LPS-CM (or ConA-CM) stimulation. Graphs were quantified with NIH Image J. **P < 0.01. **B**., **E**. Cleaved caspase-3 expression in the primary culture CGNs under treatment in the indicated conditions. **C**., **F**. IL-6 protected neurons by suppressing intracellular Ca^2+^ influx. Laser confocal scanning microscopy indicated a significant increase of intracellular Ca2^+^ concentration in neurons stimulated by the inflammatory media, LPS-CM (C) or ConA-CM (F). The effects were mostly prevented by co-culture with astrocytes, IL-6, an inhibitor of CdCl_2_ (0.2 mM), or an inhibitor of the neuron-specific MK801 (10 μM). Intracellular Ca2^+^ was labelled with the fluorescent probe Fluo-4/AM. Overall magnitude of the response is expressed as increased amplitude of intracellular Ca2^+^ transients and presented as peak normalized changes under fluorescence. L-ACM is obtained as in Figure 2E; C-ACM as in Figure 2F. Values represent group means ± SEM for four individual experiments. ***P < 0.001.

We next collected the supernatants of astrocytes after either LPS-CM or ConA-CM stimulation for 48 h, and named these media L-ACM and C-ACM, respectively. L-ACM and C-ACM contained the highest levels of IL-6 (Figure 2E and 2F). Moreover, the addition of L-ACM to the LPS-CM-stimulated neurons provided strong protection from neuronal apoptosis, yet pre-treatment with anti-IL-6 neutralizing antibodies demolished this effect in both L-ACM and astrocyte groups (Figure 3A). Neuroprotection by IL-6 was also confirmed by inhibition of cleaved caspase-3 on western blotting (Figure 3B) and by maintenance of intracellular calcium homeostasis in the neurons under stimulation (Figure 3C). Similar phenomena were observed with C-ACM in the stimulated neurons under ConA-CM treatment (Figure 3D-3F). Collectively, these data indicated that LPS-CM- and ConA-CM-stimulated astrocytes produced effective neuroprotective factors, among which the cytokine IL-6 was especially important (Figure 2C-2F and Figure 3).

We previously showed that disturbance of intracellular calcium homeostasis by inflammatory stimuli may trigger neuronal apoptosis [11]. In the present study, utilizing laser confocal scanning microscopy, we measured the intracellular Ca^2+^ concentrations ([Ca^2+^]i) in neurons stimulated with/without LPS-CM or ConA-CM, as well as with additional protective media or IL-6. We found that IL-6 and supernatants of the stimulated astrocytes protected neurons from apoptosis by suppressing intracellular Ca^2+^ influx and maintaining intracellular calcium homeostasis in neurons (Figure 3C and 3F). Thus, the apoptotic effects in neurons stimulated by inflammatory media were mostly prevented by co-cultured astrocytes, IL-6, an inhibitor of the L-type voltage-dependent calcium channel (VDC), CdCl_2_, or an inhibitor of the neuron-specific N-methyl-D-aspartate receptor (NMDAR), MK801 (Figure 3C and 3F).

### IFN-γ in the inflammatory milieu significantly promotes IL-6 secretion in astrocytes

**Figure 4:**
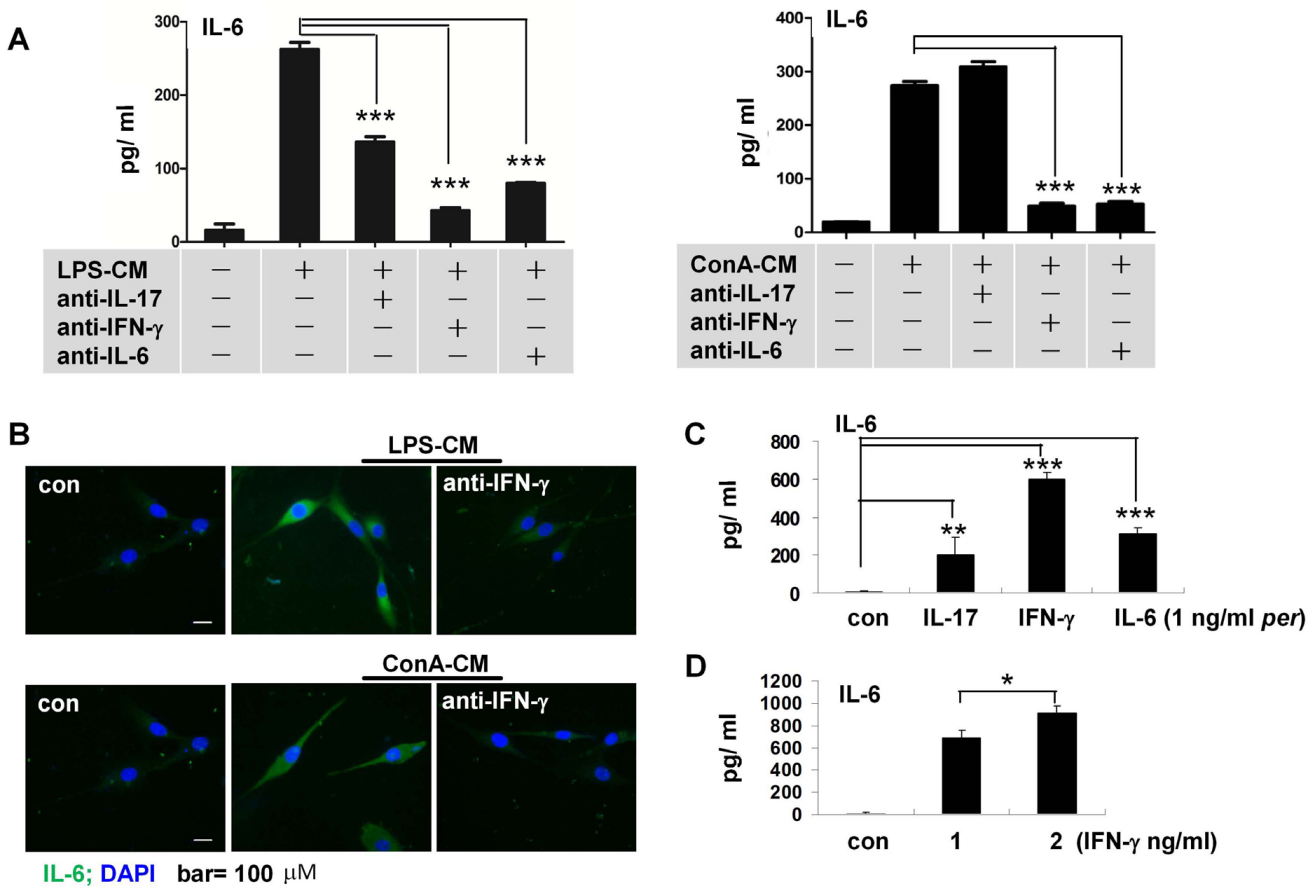
IFN-γ mediates IL-6 secretion in astrocytes stimulated by inflammatory media **A**. Anti-IFN-γ or anti-IL-6 antibodies decreased IL-6 secretion in astrocytes stimulated by either LPS-CM or ConA-CM. ELISA assays (A). Immunofluorescent staining. **B**. IFN-g stimulated IL-6 secretion in astrocytes **C**. in a dose-dependent manner **D**. Values represent group means ± SEM for four individual experiments. *P < 0.05, **P < 0.01, ***P < 0.001.

IFN-γ, IL-17, and IL-6 were present in both LPS-CM and ConA-CM. IFN-γ levels were relatively high in LPS-CM (Figure S1A); but equivalent levels of IFN-γ and IL-17, and less IL-6, were present in ConA-CM (Figure S1B). We next compared how these cytokines affected IL-6 secretion in LPS-CM- or ConA-CM-stimulated astrocytes. For this purpose, we added the respective anti-IFN-γ, -IL-17, or -IL-6 neutralizing antibodies prior to LPS-CM or ConA-CM stimulation of astrocytes, then measured IL-6 production with ELISA. The data showed that compared to IL-17, IFN-γ promoted significantly more IL-6 secretion in the stimulated astrocytes (Figure 4A). Immunofluorescence microscopy consistently showed that the astrocytes secreted much less IL-6 when we applied anti-IFN-γ neutralizing antibodies prior to either type of stimulation (Figure 4B).

We also used IFN-γ, IL-17, or IL-6 at equivalent concentrations (1 ng/ml) to challenge the astrocytes. The results showed that incubation with IFN-γ induced the highest level of IL-6 secretion in astrocytes (Figure 4C) in a dose-dependent manner (Figure 4D).

### IFN-γ induces IL-6 in activated astrocytes via STAT3 and ERK signaling pathways

**Figure 5:**
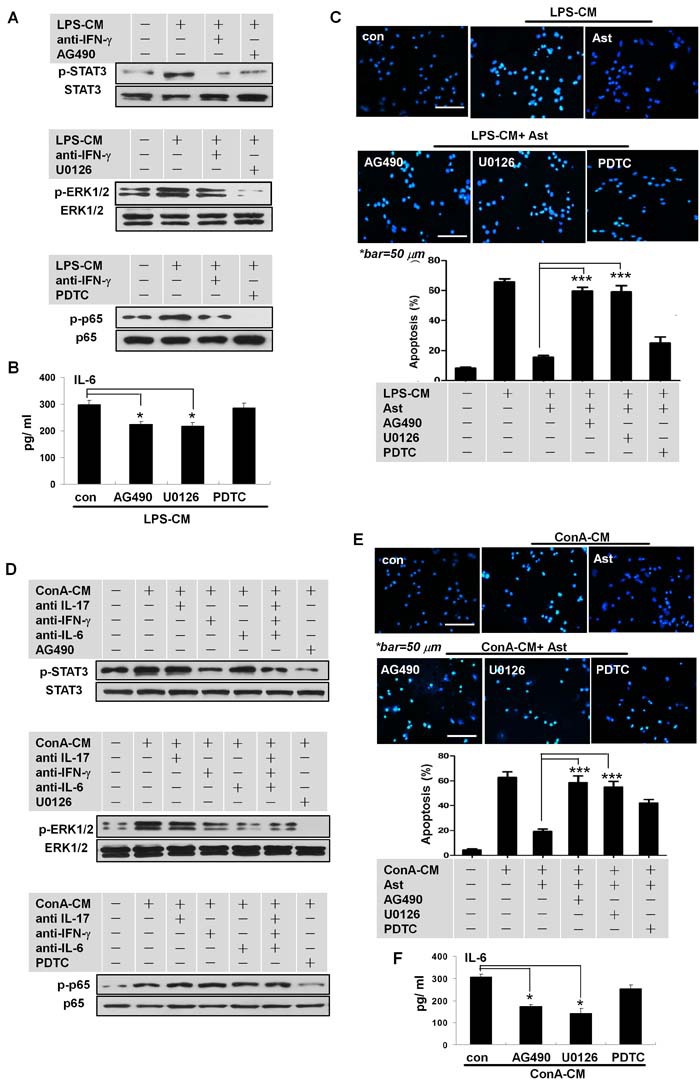
IFN-γ promotes IL-6 secretion in stimulated astrocytes via STAT3 and ERK1/2 signaling **A**., **D**. Activation of STAT3 or ERK1/2 in the stimulated astrocytes was induced by IFN-γ derived from LPS-CM or ConA-CM. **B**., **F**. IL-6 secretion in the stimulated astrocytes by LPS-CM (or ConA-CM) was inhibited by AG490 or U0126. **C**., **E**. Neuroprotection by astrocytes for neurons under LPS-CM (or ConA-CM) stimulation was abolished with AG490 or U0126 treatment. Ast: primary cultured astrocytes. *P < 0.05; ***P < 0.001.

We next set out to determine specific intracellular signaling pathways in the astrocytes stimulated by the inflammatory milieus to produce IL-6, especially the signaling pathways that were activated by IFN-γ. Whereas LPS-CM stimulated the activation of multiple signaling pathways, including STAT3, ERK, and NF-kB p65 (Figure 5A), pre-treatment with anti-IFN-γ neutralizing antibodies before LPS-CM stimulation of astrocytes greatly reduced STAT3 and ERK1/2 activation (Figure 5A). The inhibitors of STAT3 (AG490) and MEK1/2 (U0126), but not of NF-kB (PDTC), inhibited the secretion of IL-6 in astrocytes stimulated by LPS-CM (Figure 5B). Blockage of STAT3 activation with AG490 or of MEK1/2 with U0126, but not of p65 with PDTC, significantly reversed the protective effect of L-ACM against neuronal apoptosis (Figure 5C). These phenomena were further confirmed by the series data from the ConA-CM-stimulated astrocytes with or without respective inhibitors (Figure 5D-5F). Thus, the inflammation milieus and IFN-γ induced IL-6 production in the activated astrocytes via activation of the STAT3 and ERK1/2 signaling pathways.

### IFN-γ is neuroprotective and promotes *in situ* IL-6 secretion during acute neuroinflammation *in vivo*

To confirm these *in vitro* results, we used an acute neuroinflammation *in vivo* model with C57BL/6 mice (Figure S2A-C). This acute neuroinflammation mimicked a peripherally-derived bacterial infection [15]. In the primary model, at the mRNA level, expressions of the tested inflammatory factors in the cerebral hemisphere from mice that were intraperitoneally injected with LPS showed an acute course of inflammation, peaking at 4 h and returning to basal levels 24 h post-injection (Figure S2A (1) and S2D).

**Figure 6:**
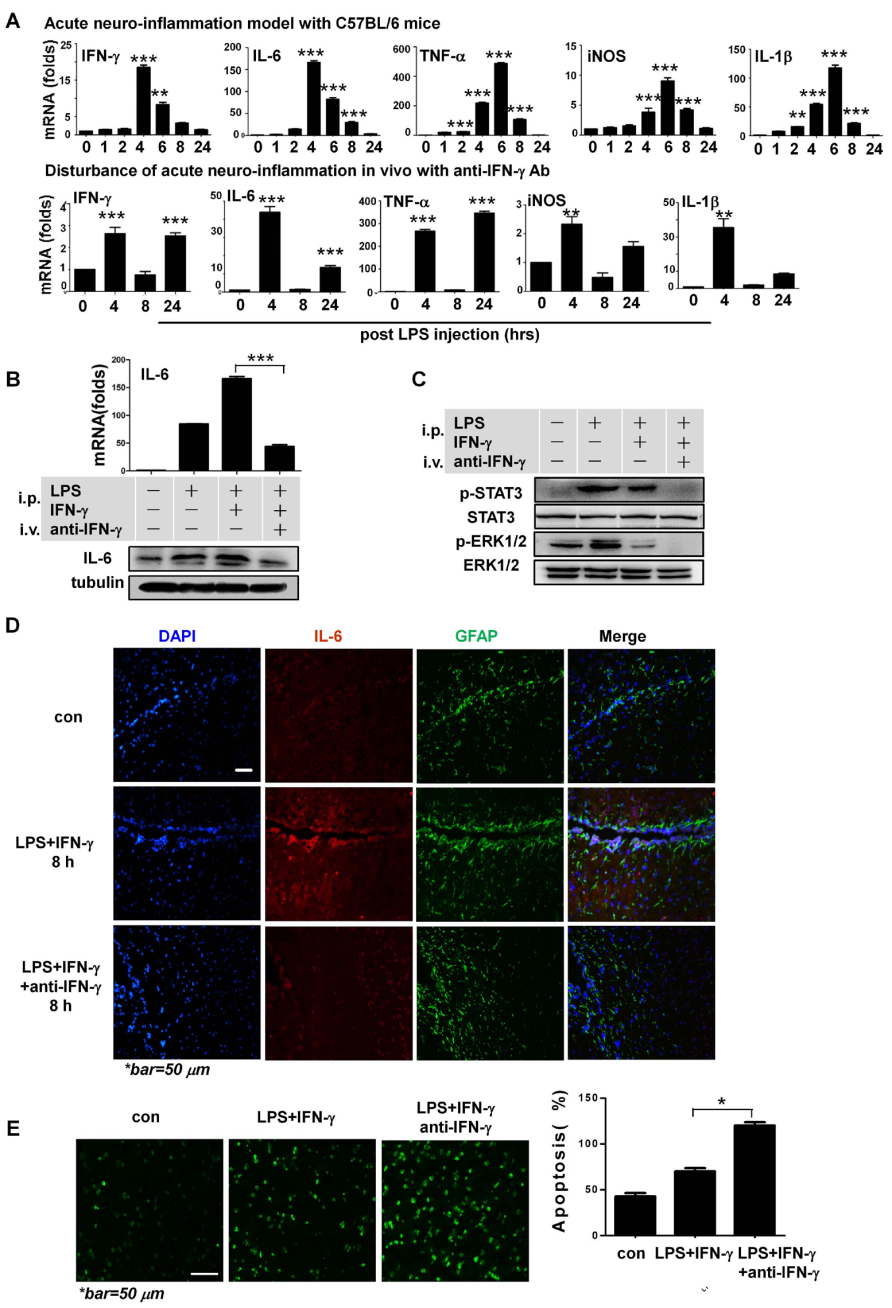
IFN-γ is neuroprotective during acute neuroinflammation *in vivo* **A**. Expressions of respective pro-inflammatory cytokines at the mRNA level during acute neuroinflammation in C57BL/6 mice as shown in Figure S2A (2), and disturbance of the acute course of disease with anti-IFN-γ antibodies in the model of Figure S2B. Note the resolved inflammation 24 h post-injection, with most of the expression levels returning to baseline in the acute neuroinflammation model without antibody treatment. **P<0.01, ***P<0.001 vs. control group. **B**. Western blotting and qRT-PCR showed IL-6 expression in CNS tissue in different acute neuroinflammation models, as shown in Figure S2. ***P<0.001. **C**. Western blotting assays indicated that STAT3 and ERK1/2 signaling were involved in IFN-γ associated functions *in vivo* during acute neuroinflammation. **D**. Immunofluorescent microscopy on cerebral cortex tissue in different groups 8 h post-LPS+IFN-γ injection in respective models Figure S2A (2) and Figure S2B. **E**. TUNEL assays and quantification of apoptosis rates of CNS tissue in models shown in Figure S2A (2) and Figure S2B 24 h post-LPS+IFN-γ injection on immunofluorescence microscopy. *P<0.05. All group data are expressed as mean ± SEM of at least three experiments. One- or two-way ANOVA was applied where appropriate, as indicated in the Materials and methods section.

To clarify the role of IFN-γ in acute disease, we co-injected recombinant IFN-γ along with the LPS to stimulate acute inflammation (Figure S2A (2)). As expected, while still following an acute neuroinflammation pattern, each of the tested inflammatory factors were significantly more strongly expressed in the co-injection model (Figure 6A upper panel, and Figure S2D). Moreover, anti-IFN-γ antibodies (intravenous) significantly disturbed the acute course of neuroinflammation. Specifically, expression levels of the tested factors did not return to normal by 24 h post-injection (Figure 6A lower panel), and their respective levels of expression were much lower during the disease, compared to the model without antibody treatment (Figure 6A). Collectively, these results showed that IFN-γ strongly promoted the production of other cytokines, including IL-6, and played a key role in determining the course of neuroinflammation *in vivo*.

Western blotting assays confirmed significantly decreased levels of IL-6 with the use of anti-IFN-γ antibodies (Figure 6B), and decreased the IFN-γ-associated signaling *in vivo* (Figure 6C) in different models 4 h after the injection of LPS plus IFN-γ. Data from immunofluorescent microscopy indicated that at the peak of neuroinflammation at 8 h (or 4 h) after LPS+IFN-γ injection, glial fibrillary acidic protein (GFAP)-expressing astrocytes secreted IL-6 (Figure 6D, Figure S3). At 24 h after LPS+IFN-γ injection in the antibody treatment group, the rates of neurocyte apoptosis were significantly higher (Figure 6E) and expression of the tested inflammatory cytokines did not return to basal levels (Figure 6A lower panel). These *in vivo* data were highly consistent with the *in vitro* data.

## DISCUSSION

This study used *in vitro* and *in vivo* acute neuroinflammation models to elucidate novel mechanisms of neuroprotection, in particular how the pro-inflammatory cytokines IFN-γ and IL-6 provide neuroprotection via astrocyte-associated functions. We recently reported on the role of pro-inflammatory IL-17 in similar scenarios [11]. However, these mechanisms and functions may be specific to the environment and stage of inflammation.

CNS tissue is composed mostly of neurons and astrocytes; the latter actively provide support, protection, and nutrients to maintain neuronal function. Depending on the situation, astrocytes may protect or damage neurons [16–18]; substantial evidence supports a neuroprotective role [19–25]. Upon encountering detrimental stimuli, astrocytes activate quickly and release factors that are potentially protective against neuronal damage via complex underlying mechanisms [26, 27]. Cytokines produced by astrocytes in an acute neuroinflammatory environment, including IL-6, are neuroprotective [26, 28]. Fujishita et.al showed that IL-6 by itself had a neuroprotective effect on neurons [29]. Research has indicated that GFAP-expressing astrocytes are activated and provide neuronal protection via ERK [30] and/or STAT3 [31] signaling during inflammation. Other studies have suggested that an intricate regulatory system controls inflammation between neurons and glial cells via cell-to-cell contact rather than with diffusible factors from neurocytes [32]. The present study showed that, in addition to the secreted IL-6, the cell-to-cell communication between neurons and astrocytes also assured neuroprotection in our acute inflammation model.

Pro-inflammatory cytokines, including IFN-γ, TNF-α, IL-6, and IL-1β, are actively produced during the immune response to fight against pathogenic infection. IFN-γ is produced mainly by activated T cells and NK cells, and plays a key role in the immune defense against invading pathogens and in immune surveillance against tumor development [33]. Whereas previous reports showed the effectiveness of IFN-γ against bacterial or viral neuroinfections [34], the role of IFN-γ in neuroprotection is not completely clear and seems indirect [35]. For example, a few reports showed that IFN-γ may help neurons resist neurodegeneration [36–38]. Consistent with our data, IFN-γ played a unique role in neuronal recovery via stimulation of glial cells [39–41] through the production of neurotrophic factors [42]. Improved neurogenesis due to microglial activation was also reported to be related to the number of T cells migrating to the hippocampus [38, 43, 44]; severely immunodeficient mice lacking functional T cells showed reduced neurogenesis [43]. Thus, T cell-derived IFN-γ plays an important role in neurogenesis.

The immune system and organs act in coordination to protect the body against a multitude of pathogens [45]. While acute inflammation is necessary to fight off pathogenic infections, it may also potentially cause neuronal damage. Astrocytes may buffer the inflammatory environment by providing neuroprotection while also maintaining the planned amplitude and timing of inflammation, while homeostasis is gradually restored [1, 2, 21]. A disturbance of the necessary course of acute inflammation may be detrimental [42]. Our data showed that treatment with anti-IFN-γ neutralizing antibodies to suppress cytokine expression during inflammation disturbed the balance and led to unresolved inflammation in C57BL/6 mice; in our previous report, anti-IL-17 neutralizing antibodies had a similar effect on acute uveitis in Lewis rats [11]. During the course of acute inflammation, many factors and cytokines are expressed in a coordinated manner. We proposed that these factors may function as a network, with IL-17 and/or IFN-γ playing key roles, because inhibiting IFN-γ levels with the specific antibody simultaneously suppressed the levels of other factors, including IL-6, *in vivo* (Figure 6). Acute inflammation is overall neuroprotective and beneficial for removing infectious threats and maintaining homeostasis. Understanding the mechanisms of acute inflammation may help to elucidate the altered regulation observed in chronic inflammation and disease. High-throughput technology makes it easier to recognize the patterns of multiple factors during inflammation and disease and how they may transform pathological situations to homeostasis in due course.

## CONCLUSIONS

This study demonstrated that acute-inflammation-induced neuronal apoptosis can be inhibited by reactive astrocytes, with IL-6 as a key neuroprotective factor. IFN-γ is involved in the neuroprotection process via activation of the STAT3 and ERK pathways in activated astrocytes. Our findings may provide novel insights into the mechanisms of immune privilege and the immune response during acute neuroinflammation, and may impact immune-regulation-based therapies for inflammation-related CNS injury and disease.

## MATERIALS AND METHODS

### Chemicals, antibodies, cytokines, and inhibitors

Lipopolysaccharide (LPS), concanavalin A (ConA), and protein inhibitors, including AG490 (for JAK2/STAT3), U0126 (for MEK1/2), and PDTC (for NF-kB), were purchased from Sigma (St Louis, MO, USA). Recombinant rat IL-6, IFN-γ, and anti-IL-6 antibodies were from PeproTech (Rocky Hill, NJ, USA). Recombinant rat IL-17 was from ProSpec (Ness-Ziona, Israel) and anti-IL-17 antibody was from Acris (San Diego, CA, USA). Anti-IFN-γ antibody was from Biosource (Camarillo, CA, USA). Other antibodies (including anti-cleaved caspase 3 antibody) were purchased from Cell Signaling Technology (CST, Danvers, MA, USA).

### Primary astrocyte and neuron cultures and co-cultures

The cultures of cortical astrocytes and cerebellar granule neurons (CGNs) were performed as described previously with minor modifications. In brief, the cerebral cortex and cerebellum were dissected from newborn or 3-day-old Wistar rats, and the meninges and blood vessels were carefully removed under sterile conditions. The neural tissues were digested with 0.25% trypsin/0.53 mM EDTA at 37 °C for 25 min. The primary astrocytes were cultured in Dulbecco's modified Eagle's medium (DMEM, GIBCO Life Tech, Paisley, UK) supplemented with 10% fetal bovine serum (FBS, GIBCO), 100 U/mL of penicillin, and 0.1 mg/mL of streptomycin, then plated on 6-well plates at a density of 5×10^5^ cells/well. The astrocytes were passaged every 2–3 days and used after 5 passages. The neurons were plated at a density of 4×10^5^ cells/well in 12-well plates coated with 25 μg/mL poly-D-lysine (Sigma), and cultivated in basal medium Eagle (BME, GIBCO) supplemented with 25 mM KCl and 2 mM glutamine (GIBCO), and complemented with 2% B27 (GIBCO). The reagent 1-p-D-arabinofuranosylcytosine (2 mg/ml; Sigma) was added to the CGN cultures 24 h after plating to limit the growth of non-neuronal cells [11]. The CGNs were used for experiments after 5 days in the culture. The purity of the primary-cultured astrocytes and CGNs was greater than 90% on immunofluorescent microscopy with anti-GFAP antibody (Biosynthesis Biotechnology Co. Ltd, Peking, China) or neuron-specific class III beta-tubulin antibody (Tuj1) (Covance Inc., Princeton, NJ, USA) (Figure S4). For the co-culture experiments, the astrocytes were added to the CGN culture 6 h before the co-cultures were exposed to the inflammatory stimuli for further assays.

### Preparation of the conditioned media for inflammatory stimulation

Peripheral blood mononuclear cells (PBMCs) from Wistar rats were isolated with Isopaque-Ficoll (Hao Yang Ltd., Tianjin, China) density-gradient centrifugation. Freshly isolated PBMCs were cultured in RPMI 1640 (GIBCO) supplemented with 10% FBS, 100 U/mL of penicillin, and 0.1 mg/mL of streptomycin at 37 °C in 5% CO_2_. The cells (3×10^6^ cells/mL) were stimulated with LPS (100 ng/mL) or ConA (5 μg/mL) for various time periods. The stimulated cells and supernatants were harvested separately and analyzed with quantitative real-time PCR and ELISA, respectively. The supernatants of PBMCs stimulated for 24 h were used as conditioned media and named LPS-CM or ConA-CM (Figure S1). A full LPS-CM or ConA-CM medium was used to stimulate the primary cultured astrocytes, and a 1:4 diluted medium was used for CGN cultures.

### Animals and acute neuroinflammation models with C56BL/6 mice

Female Wistar rats (160–180 g, 5–6 w; newborn or 3 days old) and female C57BL/6 mice (20–25 g, 5–6 w) of specified pathogen-free grade were purchased from Peking Vital River Laboratory Animal, Ltd. (Beijing, China). All animals were fed and maintained in specified pathogen-free conditions according to the guidelines for the Care and Use of Laboratory Animals published by the China National Institute of Health. All experimental procedures were approved by the ethics committee of Harbin Medical University based on the relevant statement on research animals.

The C57BL/6 mice were randomly separated into groups of 7 mice each, and challenged with or without LPS by intraperitoneal injection (100 mL, 10 mg/mL). At 0, 4, 8, or 24 h post-injection (Figure S2A (1)), each brain was carefully removed and the cerebral cortex was dissected. One homogenized hemisphere was collected for mRNA testing and the other was immediately embedded into OCT (Optimal Cutting Temperature™) medium (Richard-Allan Scientific, Kalamazoo, MI, USA) before being snap-frozen in liquid nitrogen and stored at −80 °C until staining. Coronal sections (5 μm) were cut on the bregma plane with a cryostat (Microm HM525, Walldorf, Germany).

Additional acute neuroinflammation models with C57BL/6 mice were set up, using intraperitoneal injections of LPS in combination with recombinant IFN-γ (120 ng/mL). The CNS tissues were collected as previously described at varied time-points until 24 h post-injection (Figure S2A (2)). For groups with or without anti-IFN-γ antibody treatment (50 ng/ mL), the antibody or PBS was intravenously injected into the tail vein 2 h post-LPS injection (Figure S2B and S2C).

### Quantitative real-time RT-PCR (qRT-PCR)

The relative expressions of multiple genes at the mRNA level were examined with qRT-PCR using an ABI STEPONE Real-time PCR System (Applied Biosystem, Foster City, CA, USA). Total RNA was extracted with TRIzol reagent (Invitrogen, Carlsbad, CA, USA) according to the manufacturer's instructions. One microgram of total RNA was used for cDNA synthesis with High-Capacity cDNA Reverse Transcription Kits (Applied Biosystem). The PCR reaction was performed with the Power SYBR Green PCR Master Mix (Applied Biosystem) and the reaction procedures were as follows: an initial step at 95 °C for 10 min, 40 cycles of 95 °C for 15 s, and 60 °C for 1 min. The fold change at the mRNA level was calculated after normalization to β-actin. Values of fold changes in the control sample versus the treated samples represent averages from three or four separate experiments.

### Enzyme-linked immunosorbent assay (ELISA)

The IFN-γ, IL-6, and IL-17 secretions from PBMCs or astrocytes were measured with ELISA kits (eBioscience, San Diego, CA, USA) according to the manufacturer's instructions. All standards and samples were measured with a microplate reader (SpectraMax M5, Molecular Devices) at a wavelength of 450 nm.

### Hoechst 33342 staining and TUNEL assays for apoptosis

The CGNs or co-cultures with or without stimulation were fixed in 4% paraformaldehyde for 30 min at room temperature. After staining with 0.5 ml/cm^2^ Hoechst 33342 (Beyotime, Shanghai, China) in the dark for 15 min, the cells were observed for nuclear changes under a fluorescent microscope (Nikon ECLIPSE 80i, Tokyo, Japan) equipped with a CCD camera (Nikon DS-Ri1). The nuclei with the apoptotic characteristics of chromatin condensation, pyknosis, and/or nuclear fragmentation were counted. The apoptotic nuclei appeared bright white, in stark contrast to the blueish nuclei of non-apoptotic cells. Five visual fields (100×) were randomly selected on each slide to quantify the percentage of apoptotic cells. Each experiment was repeated at least three times. TUNEL assays were performed with the TUNEL fluorescent kit (Roche). Briefly, the frozen tissue slides were permeabilized with 0.1% Triton X-100, followed by fluorescein isothiocyanate (FITC)-labeled UTP (deoxyuridine triphosphate) staining for 1 h at 37 °C. The TUNEL-positive cells were quantified as the number of bright green spots in each photograph (100×); a total of 10 photographs were counted.

### Measurement of intercellular Ca^2+^ concentration

[Ca^2+^]i was determined as described in our previous report [11]. In brief, primary-cultured CGNs were placed on poly-D-lysine-coated 15-mm glass coverslips at a density of 5,000 cells per coverslip. On the day of imaging, neurons were washed twice with normal saline solution (NSS: 137 mM NaCl, 25 mM glucose, 10 mM HEPES, 5 mM KCl, 1 mM MgCl_2_, and 2 mM CaCl_2_ at pH 7.4). The washed cells were then labeled with 5 μM Fluo-4 AM to measure [Ca^2+^]i for 30 min at 37 °C. The neurons were washed twice with NSS and left at 37 °C for another 30 min to allow for complete hydrolysis of the acetoxymethyl (AM) ester moiety, during which time the cells were treated. The cells were treated with each of the following: recombinant rat IL-6 (200 pg/ mL), the supernatant of astrocytes stimulated by the inflammatory media for 48 h, 10 μM of MK801 (NMDA Receptor inhibitor; Santa Cruz), and 0.2 mM of CdCl_2_ (calcium-channel inhibitor; Sigma) at 37 °C for 30 min. The cells were then finally exposed to the inflammatory media and the fluorescence measurement of Ca^2+^ was captured using a laser scanning confocal microscope (Leica Microsystems, Wetzlar, Germany) at an excitation wavelength of 488 nm and an emission wavelength of 530 nm for [Ca^2+^]i. The fluorescent images reflecting [Ca^2+^]i were recorded every 3.5 sec, and the data were quantified using NIH-Image J software.

### Immunofluorescence microscopy

The primary-culture astrocytes were grown on glass coverslips. After stimulation with or without the inflammatory media for 48 h, the cells were harvested and fixed in 4% paraformaldehyde and blocked in blocking buffer at room temperature for 1 h, followed by incubation in the primary anti-rat IL-6 (PeproTech) or rabbit anti-mouse IL-6 (Bioss Ltd., Beijing, China) or anti-GFAP antibodies at 4 °C overnight. The cells were then incubated with the FITC-conjugated goat anti-mouse secondary antibody (BD, Franklin Lakes, NJ, USA) or goat anti-rabbit secondary antibodies (red, Beyotime Ltd., Shanghai, China) at room temperature for 1 h in the dark. The cells were then incubated with 2 μg/mL of 2-(4-amidinophenyl)-6-indolecarbamidine dihydrochloride (DAPI; Roche) for 10 min before the slides were mounted. The images were obtained and analyzed with a fluorescence microscope (Nikon 80i) with a cold CCD camera (Nikon DS-Ri1) and NIS-Elements F 3.0 software. For cryostat sections, two independent observers evaluated 30 optical fields for every one of 10 sections per animal (7 animals per group) with a qualitative scale.

### Western blotting analysis

The neurocytes or tissues were lysed and the soluble supernatant was used for western blot analysis. Fifty micrograms of protein were separated by 10–12% sodium dodecyl sulfate-polyacrylamide gel electrophoresis (SDS-PAGE) and transferred to a PVDF membrane using a semi-dry transfer apparatus (BD). After blocking nonspecific binding with 5% nonfat dry milk, the membranes were incubated with the primary antibodies specific for cleaved caspase-3, phospho-STAT3, STAT3, phospho-ERK1/2, ERK1/2, phospho-p65, p65, and actin (Sigma) at a 1:1000 dilution at 4 °C overnight. The membranes were then incubated in peroxidase-conjugated secondary antibody (Sigma) at a 1:1000 dilution for 1 h at room temperature and developed with an ECL system (Roche), and the levels of proteins and phospho-proteins were quantified by densitometry with NIH-Image J. The antibodies were from Cell Signaling Tech. (CST) unless otherwise specified.

### Statistical analysis

All experiments were repeated at least three times. All group data are expressed as mean ± SEM. Group means were compared using one- or two-way ANOVA with treatment as the independent variable. When ANOVA showed a significant difference, pairwise comparisons between group means were examined with post hoc analysis using the Newman-Keuls multiple-comparisons test. Statistical analysis was performed using GraphPad Prism version 6.00. Differences were considered significant when the *p* value was <0.05.

## SUPPLEMENTARY MATERIALS FIGURES AND TABLES



## Abbreviations

AD: Alzheimer disease
ANOVA: Analysis of Variance
CNS: nervous system
Con A: Concanavalin A
ELISA: encephalomyelitis Enzyme-linked immunosorbent assay
GFAP: glial fiber acidic protein
IFN: Interferon
IL: Interleukin
LPS: Lipopolysaccharide
NO: Nitric oxide
PBMC: Peripheral blood mononuclear cells
PCR: Polymerase chain reaction
TNF: Tumor necrosis factor
