# Supplementary material for: Neuroprotection by IFN-γ via astrocyte-secreted IL-6 in acute neuroinflammation
Source: Oncotarget. 2017 Apr 9;8(25):40065–78. doi: 10.18632/oncotarget.16990 (PMC5522245; doi:10.18632/oncotarget.16990)
Supplement: Supplementary file 1 [file oncotarget-08-40065-s001.pdf]

Neuroprotection by IFN-γ *via* astrocyte-secreted IL-6 in acute neuroinflammation

Supplementary Material

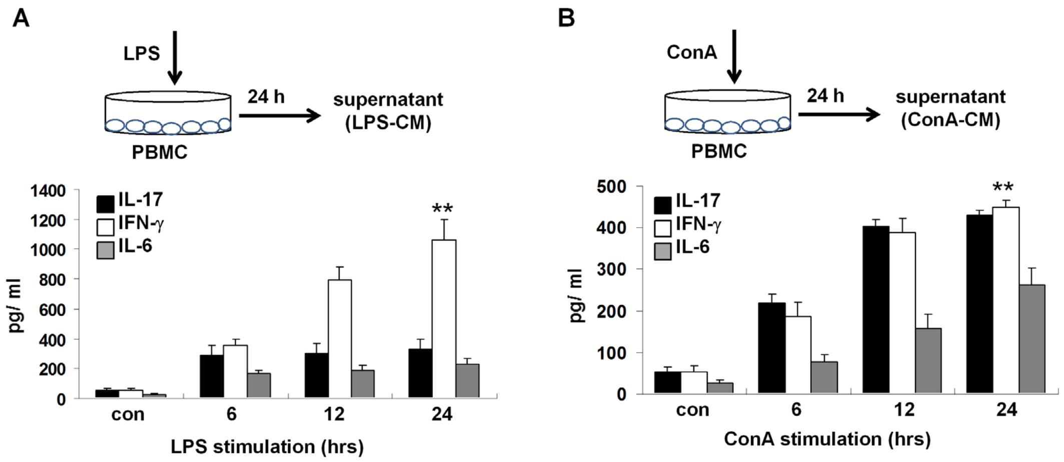

Figure S1 The inflammatory media LPS-CM and ConA-CM Peripheral blood mononuclear cells (PBMCs) of Wistar rats were stimulated with LPS (100 ng/mL) or ConA (5 μg/mL) for varied time. The cytokine secretion in the collected supernatant was analyzed by ELISA. The supernatant of the PBMCs stimulated for 24 h was used as the conditioned medium and named as LPS-CM (A) or ConA-CM (B).**, $P < 0.01$.

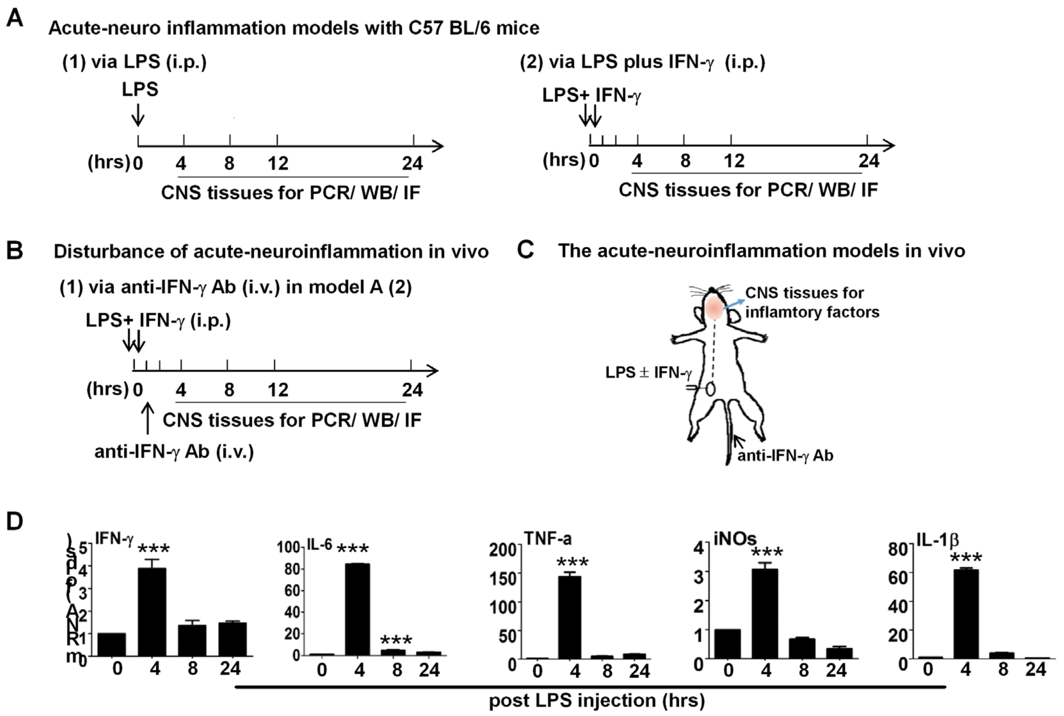

Figure S2 Induction of acute neuroinflammation models with C57BL/6 mice (A) Schematic representation of the experimental models of acute neuroinflammation with C57BL/6 mice by intraperitoneally injection of LPS (1) and LPS+IFNγ (2). (B) Schematic representation of the acute neuroinflammation model with C57BL/6 mice by intraperitoneally injection of LPS+IFNγ (2) and the treatment with anti-IFN-γ neutralizing antibodies. (C) Schematic representation of the induction of the acute neuroinflammation model with C57BL/6 mice with or without antibody treatment. (D) The expressions of respective pro-inflammatory cytokines at the mRNA level during the course of an acute neuroinflammation model with C57BL/6 mice as shown in Fig. S2A (1) *** $P < 0.001$ vs con.

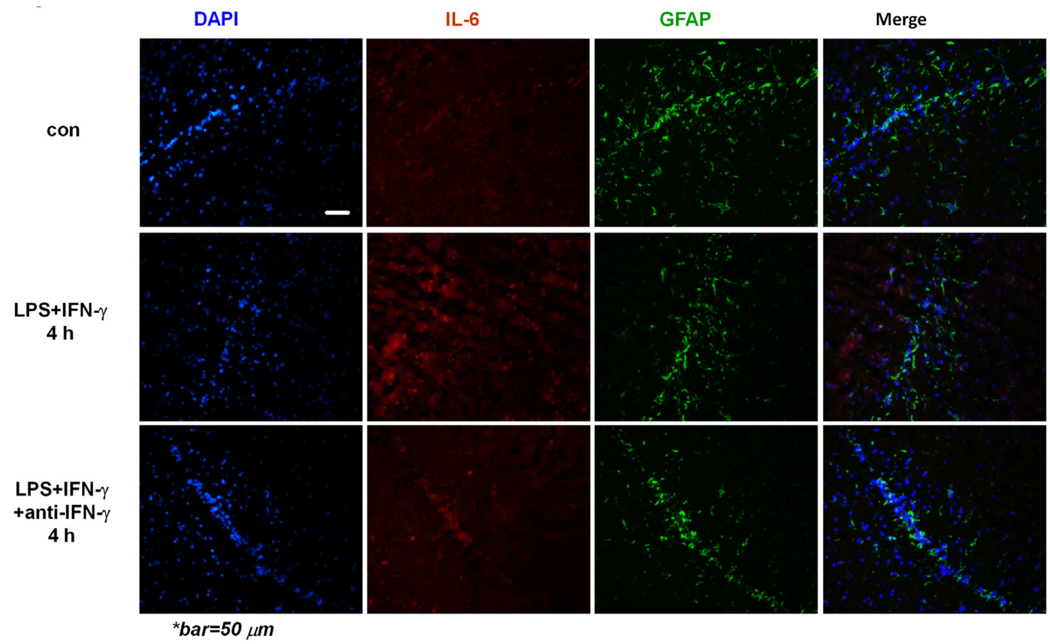

Figure S3 IL-6 secretion in the CNS tissue of acute neuroinflammation models with or without anti-IFN-γ antibody treatment Immunofluorescent microscopy on the expression of GFAP as the marker for astrocytes and IL-6 in the CNS tissue at 4 h post LPS+IFN-γ injection during acute neuroinflammation in models Fig. S2A(2) and Fig. S2B. * All group data are expressed as mean ± SEM of at least three experiments. One- or two-way ANOVA was applied where appropriate as indicated in Materials and methods section.

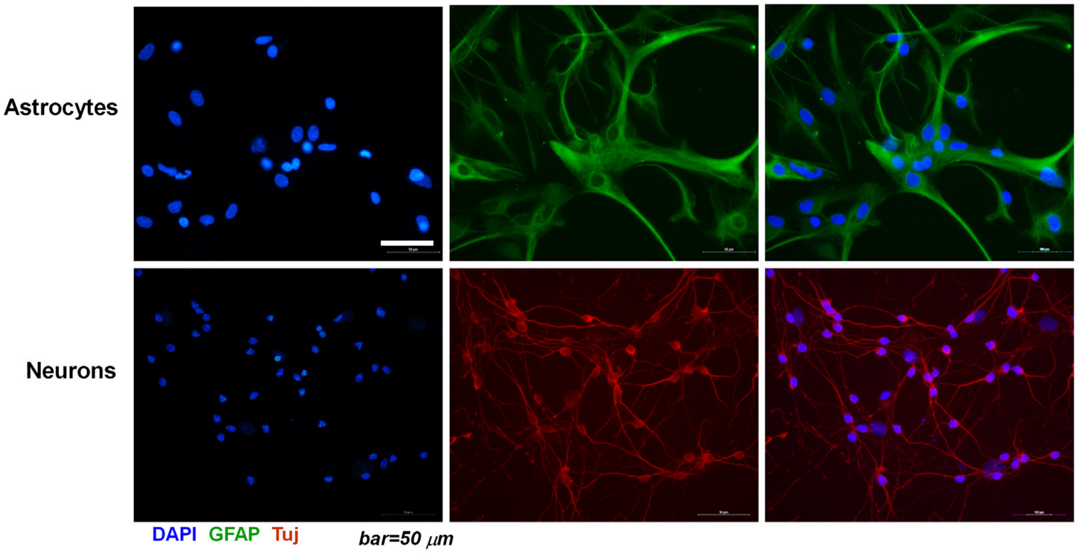

Figure S4 The primary cultures neurons and astrocytes Confirmation of the purity of the primary cultured cerebral astrocytes by anti-GFAP antibody staining and anti- beta-tubulin antibody (Tuj1) staining by immunofluorescent microscopy. Results are representative of at least three repeated experiments.

Supplemental Table 1

Results on the relative expression of immune-related factors at the mRNA level in Rat PBMC stimulated with LPS for 24 h by qRT-PCR.

	CT value	0h	6h	12h	24h
IL-17	37.15	1.00	35.41	52.94	2.81
IFN-γ	34.3	1.00	466.52	54.11	30.38
IL-6	26.92	1.00	287.19	41.50	16.38
IL-12P35	35.89	1.00	30.70	29.65	18.77
IL-22	36.46	1.00	30.91	0.62	0.16
IL-23P19	32.31	1.00	7.46	6.73	0.79
NGF	34.93	1.00	4.03	32.45	2.39
TGF-B	27.94	1.00	28.25	0.91	26.54
CNTF	29.93	1.00	32.22	35.75	19.29
T-bet	25.93	1.00	2.16	0.99	0.69
ROR-rt	31.92	1.00	71.01	32.67	10.85
Foxp3	28.93	1.00	6.63	1.13	0.56
GATA3	24.92	1.00	0.78	0.50	0.31

Supplemental Table 2

Results on the relative expression of immune-related factors at the mRNA level in Rat PBMC stimulated with ConA for 24 h by qRT-PCR.

	CT value	0h	6h	12h	24h
IL-17	37.04	1.00	50.26	0.49	11.61
IFN-γ	32.67	1.00	49.74	0.74	1.43
IL-6	36.92	1.00	42.13	5.64	7.59
IL-12P35	35.89	1.00	10.93	1.97	10.06
IL-22	36.46	1.00	9.19	0.90	4.17
IL-23P19	32.31	1.00	8.34	1.62	2.41
NGF	34.93	1.00	9.65	6.32	3.36
TGF-B	27.94	1.00	30.48	8.46	13.55
CNTF	29.93	1.00	0.42	1.46	1.06
T-bet	29.57	1.00	1.91	0.26	0.54
ROR-rt	37.23	1.00	948.83	955.43	388.02
Foxp3	35.94	1.00	32.22	12.38	18.51
GATA3	27.59	1.00	1.12	0.38	0.52

* data are representative of at least three experiments.
